# Interventions for Alcohol Use and Alcohol Use Disorders in Youth

**Published:** 2004

**Keywords:** adolescent, alcohol abuse, alcohol dependence, AOD (alcohol and other drug) use pattern, diagnostic criteria, biological development, psychological development, environmental-level prevention, individual-level prevention, family intervention, school-based intervention, brief intervention, Project Northland

## Abstract

Designing effective interventions for adolescents with alcohol use disorders (AUDs) presents several challenges, not the least of which is the accurate diagnosis of these disorders. Diagnostic criteria for AUDs have been derived largely from clinical and research experience with adults. When these criteria were tested among adolescents, numerous developmental differences were found that may affect the applicability of AUD criteria to this age group. Despite the absence of clear diagnostic criteria for use with adolescents, research has identified interventions that show promise for use with youth. This article examines both environmental- and individual-level approaches to underage drinking prevention, including school- and family-based programs, and macroenvironmental and multicomponent comprehensive interventions. Finally, it describes brief and complex treatment interventions.

## Overview

The ultimate goal of research on drinking by youth is to reduce the rates of drinking by adolescents and successfully treat those who develop problems linked to alcohol use. Prevention efforts may be aimed at keeping adolescents from starting to drink or at preventing the escalation of drinking and negative consequences. Research can provide the science on which to base the design of interventions and the means for determining which interventions are effective.

A valid diagnostic system is essential for assessing the nature and magnitude of adolescent problem drinking. Existing diagnostic criteria are derived largely from experience with adults, but developmental differences in alcohol use patterns suggest the need to adapt criteria to make them relevant and informative for an adolescent’s stage of maturation.

Prevention efforts approach the issue of youth drinking in two ways: Environmental-level interventions seek to reduce the availability of alcohol to youth and opportunities to drink, increase penalties for violation of minimum legal drinking age laws, and reduce community tolerance for alcohol use by youth. Individual-level interventions seek to change knowledge, attitudes, and skills so that youth are better able to resist influences that support drinking.

In their efforts to reduce adolescent drinking, schools and families can act at both the environmental and the individual level. School curricula operate at the individual level by trying to provide students with the knowledge, skills, and motivation to resist pressures to drink. At the environmental level, schools can make changes to discourage violation of alcohol rules and engage students’ involvement in their schools, a factor that has been found to predict less alcohol and other drug involvement.

The ability of parents to influence whether their children drink is well documented and is consistent across racial/ethnic groups. Family interventions encourage parents to be aware of the risks from underage drinking, communicate with children, clarify expectations, set rules and consequences about alcohol use, and monitor children’s activities. In addition to changing the knowledge and skills of young people, families can create an environment that reduces alcohol availability and increases the costs associated with drinking.

Research is providing data on the effectiveness of school- and family-based intervention programs and the elements that successful programs incorporate. One goal of continuing research is to improve investigators’ ability to measure outcomes and to compare studies and the methods they use as a means of changing adolescent behavior.

Community-level environmental interventions include strategies such as implementing restaurant/bar server training, checking alcohol vendors for compliance with underage laws, deterring adults from purchasing alcohol for minors, strengthening policies to detect and stop underage drinking parties, and instituting publicity for policies aimed at enforcement of laws against driving under the influence (DUI) and underage drinking. Community prevention trials have demonstrated that such efforts can reduce alcohol-impaired driving and fatal crashes among underage drivers and sales of alcohol to minors.

The most comprehensive interventions encompass coordinated school, family, and community programs. One such universal prevention program, Project Northland, was tested in 22 school districts in northern Minnesota in a randomized trial. The intervention included school curricula, peer leadership, parental involvement programs, and communitywide efforts to address community norms and alcohol availability. The intervention was delivered to a single cohort from grades 6 through 12. Comparisons in such measures as “tendency to use alcohol” and drinking five or more drinks in a row revealed differences between intervention and comparison communities.

Although the Project Northland intervention was able to reduce rates of drinking among students who were nondrinkers at the start of the project, the effort had no effect on those who already had been drinking. These very early starters are likely to have particular risk factors that make them more likely to drink and less likely to respond to more broadly targeted interventions; the experience with Project Northland suggests that programs may be needed that are aimed specifically at this group.

Underscoring the need for effective means of prevention are 2002 prevalence data indicating that, among youth ages 12 to 17, 1.4 million met the criteria set forth in the *Diagnostic and Statistical Manual of Mental Disorders, Fourth Edition* (DSM–IV) for alcohol abuse and dependence (Substance Abuse and Mental Health Services Administration [SAMHSA] 2003). The data, moreover, reveal a major unmet need for treatment for alcohol and related behavioral problems. Only 227,000 of the youth meeting criteria for alcohol problems received any treatment for these disorders in 2002. Data on alcohol problems among youth also may understate the prevalence of these disorders; alcoholism treatment researchers believe that DSM–IV criteria need to be developmentally specific to adequately identify youth with problems.

Adolescents in treatment for alcohol use disorders (AUDs) are likely to have more than one substance use disorder and may have other psychiatric comorbidities; the success of treatment is lower with those who have multiple problems than with other subgroups of youth. To date, treatment for adolescent addiction has involved adapting adult treatments to youth. Ongoing research is testing some innovative and developmentally tailored interventions aimed at improving treatment outcomes.

Some of the most promising interventions for adolescents with AUDs have been complex, multicomponent therapies. The current health care financing system stresses the need for shorter, more cost-effective treatment, however. An alternative to complex treatments, brief interventions can be directed at drinking or the consequences of drinking. An example of a brief intervention is motivational enhancement, which encourages the person to take responsibility for change and provides a menu of options for change. Early evidence suggests that brief interventions can be helpful in reducing both drinking and its consequences in adolescents.

Overall, research points to the importance of applying a more nuanced and detailed understanding of adolescent development to the design of treatments and outcome measures for alcohol use problems in adolescents.

## Diagnosis of Alcohol Abuse and Dependence in Adolescents

A valid diagnostic system is essential to advancing treatment and research of adolescent AUDs. Diagnoses should facilitate communication among clinicians and researchers, identify cases for different levels of clinical intervention, provide phenotypes for genetics research, and convey information about prognosis (Robins and Barrett 1989; McGue 1999). DSM–IV (American Psychiatric Association [APA] 2000) includes two AUDs, alcohol abuse and alcohol dependence, which are defined by nonoverlapping criterion sets. DSM–IV abuse focuses on negative psychosocial consequences resulting from drinking, as well as hazardous use, and requires the presence of at least one of four criteria. DSM–IV dependence is diagnosed when at least three of seven criteria related to physical dependence, salience of alcohol use, and impaired control over drinking behavior are met within the same 12-month period. Both DSM–IV AUDs require evidence of clinically significant impairment or subjective distress resulting from alcohol use for diagnosis.

Diagnostic criteria for AUDs were derived largely from clinical and research experience with adults, and only recently has their validity been assessed among adolescents (Chung et al. 2005). Numerous developmental differences between adolescents and adults may affect the applicability of AUD criteria to youth. For example, adolescents tend to drink less often than adults but typically consume a greater quantity per occasion (Deas et al. 2000). Developmental differences in alcohol use patterns indicate the need to adapt criteria to make them relevant to and properly scaled for an adolescent’s stage of maturation (Brown 1999). Because a construct may manifest itself differently in adolescents and adults (e.g., role impairment at school vs. work), a perspective that takes developmental factors and contextual influences into account is essential for valid assessment of AUD symptoms.

DSM–IV AUDs have shown some validity when used with adolescents in that teens classified as having alcohol dependence, abuse, and no diagnosis differ on external measures of alcohol involvement (e.g., Lewinsohn et al. 1996; Winters et al. 1999). Several important limitations have been identified, however, both at the criterion level of how symptoms are defined and measured and at the level of the diagnostic algorithms for alcohol abuse and dependence. At the criterion level, certain symptoms (e.g., withdrawal, use despite medical problems) tend to occur only after years of heavy drinking and have low prevalence and limited utility when applied to teens. Other DSM–IV AUD symptoms appear to be more relevant to specific adolescent subgroups. For example, hazardous use and legal problems have been associated with male gender, increased age, ethnic background, and presence of conduct disorder symptoms in teens (Langenbucher and Martin 1996; Wagner et al. 2002). Ethnicity and gender have been found to influence whether and when certain DSM–IV AUD symptoms tend to occur in teen drinkers (Wagner et al. 2002).

**DSM–IV Diagnostic Criteria for Alcohol Abuse and Dependence**

***Alcohol Abuse***

(A) A maladaptive pattern of drinking, leading to clinically significant impairment or distress, as manifested by at least one of the following occurring within a 12-month period:

- Recurrent use of alcohol resulting in a failure to fulfill major role obligations at work, school, or home (e.g., repeated absences or poor work performance related to alcohol use; alcohol-related absences, suspensions, or expulsions from school; neglect of children or household)
- Recurrent alcohol use in situations in which it is physically hazardous (e.g., driving an automobile or operating a machine when impaired by alcohol use)
- Recurrent alcohol-related legal problems (e.g., arrests for alcohol-related disorderly conduct)
- Continued alcohol use despite having persistent or recurrent social or interpersonal problems caused or exacerbated by the effects of alcohol (e.g., arguments with spouse about consequences of intoxication).

(B) Never met criteria for alcohol dependence.

***Alcohol Dependence***

(A) A maladaptive pattern of drinking, leading to clinically significant impairment or distress, as manifested by three or more of the following occurring at any time in the same 12-month period:

- Need for markedly increased amounts of alcohol to achieve intoxication or desired effect; or markedly diminished effect with continued use of the same amount of alcohol
- The characteristic withdrawal syndrome for alcohol (or a closely related substance) or drinking to relieve or avoid withdrawal symptoms
- Persistent desire or one or more unsuccessful efforts to cut down or control drinking; or drinking in larger amounts or over a longer period than intended
- Important social, occupational, or recreational activities given up or reduced because of drinking
- A great deal of time spent in activities necessary to obtain, to use, or to recover from the effects of drinking
- Continued drinking despite knowledge of having a persistent or recurrent physical or psychological problem that is likely to be caused or exacerbated by drinking.

(B) No duration criterion separately specified, but several dependence criteria must occur repeatedly as specified by duration qualifiers associated with criteria (e.g., “persistent,” “continued”).

Source: American Psychiatric Association (APA). *Diagnostic and Statistical Manual of Mental Disorders, Fourth Edition*. Washington, DC: APA, 1994.

Some symptoms, such as tolerance, appear to have a high prevalence among young drinkers in part because they are poorly defined or scaled for the developmental period of adolescence (Martin and Winters 1998). DSM–IV’s definition of tolerance as a “marked increase to obtain the same effect” is only modestly associated with adolescent alcohol dependence. Many adolescent drinkers report marked increases to produce the same effect (e.g., from one drink to three) but are relatively light drinkers, often not having any other symptoms. Some level of tolerance may occur as a normative developmental phenomenon in youth who drink. Other adolescents are heavy drinkers who are not assigned the tolerance symptom; they report high quantities of drinking during early drinking experiences (e.g., six or more drinks) without a subsequent marked increase to produce the same effect (Chung et al. 2001). Better guidelines need to be developed regarding the identification of clinically significant levels of tolerance in teens, or alternatives such as a heavy drinking criterion must be considered (Chung et al. 2001).

Some AUD criteria may be interpreted differently or have different meanings when used with adolescents compared with adults, such as “drinking more or longer than intended.” This symptom often is assigned as a result of an adolescent’s poor judgment, inexperience with alcohol’s effects, or social pressures to drink, rather than as a compulsive pattern of alcohol use (Chung and Martin 2005). Research has examined the development of more specific interview probes that query contextual factors, such as adolescents’ motivations for drinking and reasons for limiting alcohol use, as a way to increase the validity of this symptom among youth. Differences in how tolerance and drinking more or longer than intended are assessed affect diagnostic validity and have a large effect on the estimated prevalence of AUDs in adolescent community samples (Chung et al. 2002).

There are other limitations of the DSM–IV at the level of diagnostic algorithms, that is, abuse as one out of four criteria and dependence as three out of seven criteria. Some adolescents who engage in relatively low levels of alcohol use meet criteria for an abuse diagnosis only because of arguments with their parents about alcohol use and may be considered to be “diagnostic impostors” (Martin 1999). However, “diagnostic orphans,” who have one to two dependence symptoms and no abuse symptoms, and thus no DSM–IV AUD, are similar to teens with DSM–IV alcohol abuse on drinking levels and clinical outcomes (Pollock and Martin 1999). Diagnostic impostors and orphans limit the ability of the DSM–IV diagnostic system to provide appropriate categories for research studies and to guide the allocation of scarce health care resources.

The DSM–IV’s separate criterion sets for abuse and dependence are not well distinguished conceptually or empirically. Data do not support a distinction between the two categories in severity, age of symptom onset, age of onset of the two diagnoses, or symptom profiles identified by latent class analysis and factor analysis (e.g., Martin et al. 1996; Wagner et al. 2002). Some community surveys report higher prevalence of the more severe dependence diagnosis relative to the milder abuse diagnosis (Chung et al. 2002), a situation that does not conform to most disorders in psychiatry or medicine. In contrast to the DSM–IV dichotomy of abuse and dependence, evidence suggests that the latent structure of adolescent alcohol problems represents a continuum of severity distinguished more by the number than the type of symptoms (Chung and Martin 2001).

Longitudinal studies indicate that alcohol problems which occur in adolescence and young adulthood are only modestly associated (e.g., Baer et al. 1995; Rohde et al. 2001). The alcohol abuse diagnosis appears to be particularly transient, with a high rate of transitions into and out of this category (Nelson and Wittchen 1998). Many adolescents with AUDs mature out of problem drinking (Labouvie 1996; Maisto et al. 2002), whereas others show a more chronic course through adulthood (Abrantes et al. 2002). Multiple developmental trajectories of adolescent-onset AUDs exist (e.g., Schulenberg et al. 2001) and have been characterized as developmentally limited or persistent, with diagnoses that may be relatively continuous or intermittent (Zucker et al. 1995). Ongoing longitudinal research will help investigators understand more about the clinical course and prognosis of adolescent-onset abuse and dependence and will help them test the predictive validity of diagnostic criteria, course specifiers, and algorithms in the DSM–IV and beyond.

## Prevention of Underage Drinking

### Intervention Approaches

Environmental-level interventions seek to reduce opportunities (availability) for underage drinking, increase penalties for violating minimum legal drinking age (MLDA) and other alcohol use laws, and reduce community tolerance for alcohol use and misuse by youth. Individual-level interventions seek to change knowledge, expectancies, attitudes, intentions, motivation, and skills so that youth are better able to resist the pro-drinking influences and opportunities that surround them. This section discusses four types of individual- and environmental-level programs: school-based programs, family-based programs, macroenvironmental programs, and multicomponent programs.

### School-Based Prevention Programs

School-based curricula to prevent use of alcohol and other drugs by youth have a long history. However, the use of research-based findings to guide the content and evaluation of such curricula is a fairly recent development (Bangert-Drowns 1988; Dielman 1995). The first school-based programs were primarily informational and often used scare tactics—it was assumed that if youth understood the dangers inherent in alcohol misuse, they would choose to abstain. These programs were ineffective. Better programs are now available, but researchers have found that sometimes they are not used (Silvia and Thorne 1997) or implemented as designed (Dusenbury et al. 2003).

Efforts to clarify theoretical and methodological issues relevant to improving school-based prevention curricula have made steady progress. However, methodological issues remain a critical barrier to interpreting the large number of published studies, as many were conducted with less than optimal degrees of scientific rigor. Additionally, variations in design and methodology make comparisons across studies difficult. For example, there is wide variability in alcohol use outcome measures, and it is common for some measures within a single study to show significant intervention effects whereas others do not (Foxcroft et al. 2003).

Researchers are increasingly interested in collecting information on alcohol-related problems and high-risk drinking practices in addition to more straightforward measures of quantity and frequency of drinking. Outcomes based solely on knowledge and attitudes are no longer acceptable. Variation in measures makes comparisons across studies difficult. Also, the frequent use of study-specific composite scales (based on combinations of individual measures) often makes practical interpretations of findings difficult. This latter problem, coupled with the failure to report effect sizes, makes it difficult to judge the likely benefit from implementing programs on a large scale (Gorman 1995). Analysis based on intention-to-treat is the most relevant from a public health standpoint, but application of this analytic standard often eliminates statistical significance (Foxcroft et al. 2003). Differences in program intensity (number of sessions), followup periods, age/grade of students, program goals, population characteristics, and attrition also impede meta-analysis and cross-study comparisons.

However, the following general statements are supported by the literature:

- Programs that rely primarily on increasing knowledge about the consequences of drinking are not effective.
- Effective programs often:
  – Are based on social influence models
  – Include norm setting
  – Address social pressures to drink and teach resistance skills
  – Include developmentally appropriate information
  – Include peer-led components
  – Provide teacher training
  – Are interactive.

Unfortunately, effect sizes generally are small. Even state-of-the-art programs are not sufficient to prevent adolescent use and misuse of alcohol in the absence of social and environmental change. Much of the literature suggests universal prevention curricula are less effective with higher risk students—those who have initiated drinking prior to grades five or six; additional research is needed in this area because of inconsistencies in the literature (Gottfredson and Wilson 2003; Hansen 1992; Komro and Toomey 2002; Tobler 1986; Tobler and Stratton 1997; National Research Council [NRC] and Institute of Medicine [IOM] 2004; Wagenaar and Perry 1994).

School curricula operate at the individual level by trying to provide students with the knowledge, skills, and motivation needed to resist pressures to drink. However, schools also may be considered from an environmental perspective. Policies and practices within the school, such as consistent enforcement of sanctions for violating alcohol rules, are another arena for intervention. Students’ bonding or attachment to their schools is found to predict less alcohol and other drug involvement, so overall school climate and cohesiveness also seem to be important. However, there are few studies linking specific school policies with alcohol use and even fewer studies of policy changes (Flay 2000).

### Family-Based Prevention Programs

The ability of parents to influence whether their children drink is well documented and is consistent across racial/ethnic groups (e.g., Barnes et al. 2000; Steinberg et al. 1994). Setting clear rules about children not drinking, consistently enforcing those rules, and monitoring child behavior reduce the likelihood of underage drinking. Family conflict and lack of cohesion are associated with increased risk (Bogenschneider et al. 1998). Family interventions encourage parents to be aware of the risks from underage drinking, to communicate with children, to clarify expectations regarding alcohol use, to set rules and consequences for violations, to monitor children’s activities, and to reduce the availability of alcohol in the home. Additionally, content on family management practices and communication skills often are included. Parent-directed programs have been included with school-based interventions, some of which have evidence of success; but these components have not been evaluated separately (Flay 2000). Stand-alone family interventions have been successful in reducing alcohol use and other risk behaviors (Komro and Toomey 2002).

The Iowa Strengthening Families Program (ISFP), delivered when students were in grade six, has shown long-lasting preventive effects on alcohol use, even when evaluated on the basis of intent-to-treat (Spoth et al. 2001, 2004). This finding is striking on two counts: First, it suggests that the intervention succeeded in changing the normative environment of schools in which the program was offered, because even students whose families did not participate benefited. Second, the increase in effect size over time and the duration of effects into high school compares favorably with school-based interventions. A recent Cochrane review identified the ISFP as one of two potentially effective interventions for the primary prevention of alcohol misuse by youth (Foxcroft et al. 2003).

Family interventions operate at both the individual and environmental level. Interventions seek to change behavior of both parents and children by increasing knowledge and skills. However, by changing parent practices, they affect a primary social environment for the child. This microenvironment-level change effectively reduces availability and increases “costs” associated with drinking, which probably accounts for the lasting intervention effects that have been observed.

Families in distress or youth who are exhibiting behavior problems may need more intensive interventions (selective and indicated prevention). Tiered or stepped-intervention strategies have been described to restrict more costly services to the subset of families in most need (Dishion and Kavanagh 2000; Sanders 2000).

### Macroenvironmental Interventions

Environmental approaches may have both direct and indirect influences on drinking by youth. Enforcement of MLDA laws directly reduces alcohol availability, a critical element in comprehensive risk models (Wagenaar and Toomey 2002). Penalties for alcohol use and misuse that apply directly to youth increase the social “cost” of drinking, which is expected to affect decisions about drinking. Changes in monetary price have been associated with decreases in use and related problems (Leung and Phelps 1993; Kenkel and Manning 1996; Chaloupka et al. 1998; and Cook and Moore 2002). Public awareness campaigns in support of environmental change serve to change community norms regarding the acceptability of underage drinking, which should further reduce opportunities to drink and increase social costs to young drinkers (NRC and IOM 2004; Toomey and Wagenaar 2002; Wagenaar and Perry 1994).

Environmental interventions are among the recommendations included in the recent NRC and IOM report, *Reducing Underage Drinking: A Collective Responsibility* (NRC and IOM 2004), and by the Panel on Prevention and Treatment of the National Institute on Alcohol Abuse and Alcoholism (NIAAA) Task Force of the National Advisory Council on Alcohol Abuse and Alcoholism. Such programs seek to reduce commercial and social availability of alcohol and/or reduce driving while intoxicated. They may use a variety of strategies, including implementing server training, instituting compliance checks in outlets, deterring adults from purchasing for minors (shoulder tap) or providing alcohol to minors (public education and policies), restricting drinking in public places, enforcing penalties for use of false IDs, strengthening policies to detect and terminate underage drinking parties, establishing penalties for providing alcohol to a minor, enforcing DUI and zero-tolerance laws, and creating publicity regarding policies and sanctions.

Three community trials in the United States are noteworthy and are described below. Collectively, they show the utility of community environmental strategies to reduce underage drinking and related problems.

#### The Massachusetts Saving Lives Program

This 5-year comprehensive intervention implemented in six communities was designed to reduce alcohol-impaired driving and related traffic deaths. This program decreased fatal crashes, particularly alcohol-related fatal crashes involving drivers ages 15–25, and reduced the proportion of 16- to 19-year-olds who reported driving after drinking relative to the rest of Massachusetts. It also increased teen awareness of penalties for drunk driving and for speeding. Other significant outcomes related to traffic safety were not age-specific (Hingson et al. 1996; Hingson and Howland 2002).

#### The Community Prevention Trial Program

This program was implemented in three intervention communities matched to three comparison sites. The formal goal of the project was to assist each experimental community to make effective, long-term changes to reduce alcohol-involved injuries and death but not necessarily to change individual drinking patterns. The intervention strategies included efforts to reduce alcohol availability to minors. Sales to apparent minors (people of legal drinking age who appear younger than age 21) were significantly reduced in the intervention communities compared with the control sites (Grube 1997; Holder 2000).

#### Communities Mobilizing for Change on Alcohol

This program was a randomized 15-community trial to reduce the accessibility of alcoholic beverages to youths under the legal drinking age. It emphasized environmental factors that affect the supply of alcohol to youth, using a community organizing approach to achieve policy change among local institutions. Among the significant findings were that merchants in participating communities were less likely to sell alcohol to minors and that 18- to 20-year-olds were less likely to try to purchase alcohol or provide alcohol to younger teens. There also was a decline in DUI arrests among 18- to 20-year-olds. There were no program effects, however, on self-reported drinking by 12th graders, the youngest age group surveyed. This may be a result of the short duration of the intervention—2.5 years—or it may be that younger adolescents obtain alcohol from adults and are not directly affected by changes in commercial availability (Wagenaar et al. 2000*a*,*b*).

Community-level interventions clearly can reduce commercial sales of alcohol to minors, and this can affect overall drinking by older adolescents. It remains to be seen whether sustained interventions can reduce social availability of alcohol to younger adolescents. Additionally, the fact that community interventions can simultaneously reduce alcohol-related problems among adults (e.g., injury) and youth (e.g., availability) increases their cost-effectiveness and should make them attractive to policymakers (Foxcroft et al. 2003).

### Multicomponent Comprehensive Interventions

Comprehensive interventions provide coordinated programs at the school, family, and community levels and target multiple pathways for risk. Ideally, they also should integrate universal, selective, and indicated prevention programs and treatment for youth who are alcohol dependent. To date, one such program, Project Northland, has been evaluated.

Project Northland is a comprehensive universal prevention program that was tested in 22 school districts in northeastern Minnesota in a randomized trial. The intervention included (1) innovative social behavioral school curricula, (2) peer leadership, (3) parental involvement programs, and (4) communitywide task force activities to address larger community norms and alcohol availability. The intervention was delivered to a single cohort in grades 6 through 12. Intervention intensity and focus varied over the study period. The first phase (grades 6 through 8) had strong school and family components. By the end of grade 8, fewer students had initiated alcohol use, and the prevalence of alcohol use (past month and past week) was significantly lower in the intervention communities compared with control communities (Perry et al. 1996). During the next phase of the study, grades 9 and 10, there was minimal intervention. In grades 11 and 12, intervention activities resumed, and the community component to reduce availability was featured more prominently.

Significant differences were observed between intervention and comparison communities during each project period for “tendency to use alcohol” (a composite measure that combined items about intentions to use alcohol and actual use) and “five or more in a row.” The rates of increase in underage drinking prevalence were lower in the intervention communities during phase 1; higher during the interim period (suggesting a “catch-up” effect while intervention activities were minimal); and again lower during phase 2 when intervention activities resumed (Perry et al. 2002).

Based on its success, Project Northland has been designated a model program by SAMHSA, and its materials have been adapted for a general audience and marketed by Hazelden. It now is being replicated in ethnically diverse urban neighborhoods.

### Very Early Interventions

The Project Northland findings at the same time point to a dilemma that may be a significant hurdle when working to prevent underage drinking in the highest risk groups. The program began in sixth grade, when children were approximately 12 years of age, and although it was able to reduce rates of drinking among those who were non-drinkers at the initiation of the project, the intervention had no effect on those who had already begun drinking. The study was not able to parse out the reasons for these differential effects on initial nonusers vs. users, but youth who are already drinking at sixth grade are very much an early onset group, given that the median age of onset of first use is age 14. Given also what is known about the impulsivity, heavier drinking by parents, and conflicted family backgrounds of early onset users (Ellickson et al. 2003; Mayzer et al. 2003), it is likely that the social micronetworks within which the early onset drinkers moved would have insulated them to a greater degree from the program’s effects. For this subgroup, earlier precursive risk intervention programs may be necessary (Nye et al. 1999; Spoth et al. 2001; Zucker and Noll 1987).

## Treatment for Adolescent Alcohol Use Disorders

Prevalence data on binge and heavy drinking, collected in the 2002 U.S. National Survey on Drug Use and Health (NSDUH) (SAMHSA 2003), indicate a public health problem of considerable dimensions in youth ages 12 to 17. Binge drinking is well established by midadolescence, as reported by 12 percent of 15-year-olds, 18 percent of 16-year-olds, and 25 percent of 17-year-olds. Not only are these youth at high risk for serious accidents and adverse social, health, and academic consequences related to their alcohol use, but some also may be at risk for developing multiple behavioral disorders including alcohol abuse and alcoholism. At the same time, as already discussed, alcoholism treatment researchers who specialize in youth diagnosis and treatment believe that DSM–IV criteria are inadequate to identify youth who have AUDs. They conclude that diagnosis of youth substance use disorders needs to be developmentally specific, to meet fewer criteria than required by DSM–IV, and to add criteria salient to youth drinking practices (Chung et al. 2003; Clark 2004).

**Figure:**
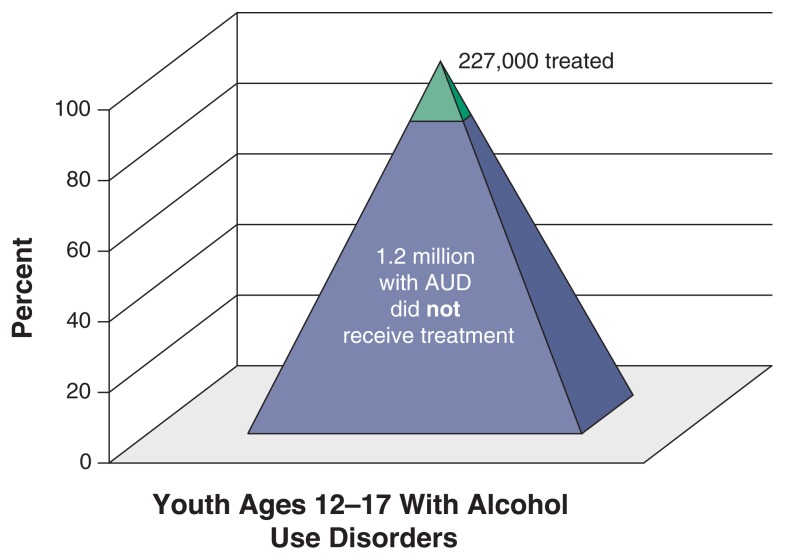
Alcohol abuse and dependence: The unmet need for treatment in youth ages 12–17, in the past year. In 2002, only 16 percent of the 1.4 million youth ages 12 to 17 estimated to have alcohol use disorders (AUDs) reported receiving any type of service for these problems. SOURCE: Samhsa, National Survey on Drug Use and Health, calculated from 2002 raw data tables available on SAMHSA Web site, http://oas.samhsa.gov/nsduh.htm.

### An Unmet Need

Nonetheless, the NSDUH data indicate a major unmet need for effective health services to prevent and treat alcohol and other associated behavioral problems. Among youth these ages, 1.4 million met DSM–IV criteria for alcohol abuse or dependence, but only 227,000 actually received any treatment for alcohol use disorders in 2002 (SAMHSA 2003). Further, current services are not optimally designed for youth access or engagement (Brown 2001). Youth prefer easy access, low threshold approaches that accentuate strategies adolescents normally use to stop drinking (Metrik et al. 2003), and treatments that do not remove them from their primary home or academic settings (Brown 2001). Youth perceive traditional services (e.g., alcoholism treatment programs, Alcoholics Anonymous) as less helpful than brief interventions tailored to salient adolescent concerns (D’Amico et al. 2004). Consequently, alternative formats, attention to developmental transitions, and social marketing are needed to more adequately address alcohol problems emerging in adolescence (Brown 2001; Kypri et al. 2004; O’Leary et al. 2002).

### Heterogeneity in Adolescents With Alcohol Use Disorders

According to 2002 NSDUH data, nicotine use and illicit drug use are much higher among drinking youth ages 12 to 17 who reported they were binge drinkers or heavy drinkers in the past 30 days than they were among those who reported drinking less. Research on adolescents in treatment for alcohol use disorders reflects a similar pattern; these youth typically use cigarettes, are likely to have more than one substance use disorder, and may manifest psychiatric comorbidities as well (e.g., Abrantes et al. 2004; Myers and Brown 1994; Rowe et al. 2004). Alcohol-dependent adolescents with psychiatric comorbidities fare more poorly after treatment: These youth have lower abstention rates, relapse more rapidly, and show deterioration in their mental health symptoms following relapse to alcohol or other drugs (Tomlinson et al. 2004; McCarthy et al. 2005). Also, adolescents in substance abuse treatment who have combined heavy alcohol use and drug disorders manifest a more severe problem profile and less successful treatment outcomes (Grella 2003). Thus, by the time many youth reach treatment, they are already on a developmental pathway that ultimately, unless deflected, could lead to even more harmful behavioral lifestyles, medical disorders, and social consequences.

It is important to keep in mind, however, that adolescents in addiction treatment are a heterogeneous group and follow multiple pathways of change post-treatment, including successful ones (Abrantes et al. 2004; Brown 2001). Results from several studies of alcohol-dependent youth consistently demonstrate that although a portion of adolescents abstain and others quickly return to problematic use after treatment, the majority of adolescents change their use patterns over time, both improving and deteriorating as they face new developmental challenges (e.g., Brown 2004). Among treated youth, alcohol use following treatment also plays a significant role in relapse to other drugs (e.g., Brown et al. 2000), as well as in functioning in school, with peers and family, and in physical and mental health (see Brown and D’Amico 2003). See Chung and colleagues (2003), “Course of Alcohol Problems in Treated Adolescents,” for analyses of (1) four longitudinal, alcohol-focused treatment outcome studies that cover 1 to 8 years post-treatment and (2) how these treatment outcomes vary by subtypes of patients, settings, and trajectories.

### NIAAA Research on Adolescent Treatment

Out of concern over the emerging evidence on the nature and magnitude of alcohol use and associated problems in underage youth, in 1997 NIAAA formally initiated a program of research to develop effective treatment interventions for adolescents with alcohol disorders. Prior to this, adult addiction treatments were extended to adolescents and rarely had been rigorously evaluated in youth (e.g., Brown et al. 2005). A total of 20 clinical projects have been funded under this NIAAA program, 14 of which were cofunded by SAMHSA’s Center for Substance Abuse Treatment. The majority of these clinical studies are randomized controlled clinical trials. The objective of this initial wave of studies is to design and test innovative and developmentally tailored interventions and, in so doing, provide evidence-based knowledge to improve treatment outcomes for adolescents who have primary alcohol use disorders or manifest at least one or two symptoms of alcohol dependence (i.e., are “diagnostic orphans” [Pollock and Martin 1999]). The results of these projects will be forthcoming over the next few years and will provide new information on the potential efficacy of family-based, cognitive-behavioral, brief motivational, and guided self-change interventions in a range of settings. They also will provide information on the efficacy of these treatments in subgroups of adolescents, including homeless and runaway youth, high school students, juvenile justice–involved youth, and minority youth. This treatment research also will shed light on distinctive features of adolescent treatment including processes of change and factors contributing to post-treatment success.

### Adolescent Treatment Interventions

Complex interventions have been developed and tested in adolescents referred for treatment of alcohol and other drug disorders. Many of these patients are likely to have more than one substance use disorder (e.g., alcohol and marijuana) and to have other psychiatric disorders as well (e.g., depression, anxiety, or conduct disorder). Brief interventions are, as a rule, delivered to adolescents in general medical settings (e.g., primary care clinics, emergency rooms) or in school-based settings. The range in severity of substance use problems encountered in the nonaddiction specialty settings is greater than in treatment centers, thereby providing the opportunity to intervene before serious social consequences and alcohol use disorders develop (Wagner et al. 1999).

#### Complex Interventions

Some of the most promising interventions for adolescents with alcohol use disorders have incorporated multiple components and systems. These include (1) family therapies with both familial and community components (i.e., multidimensional family therapy [MDFT]) (Faw et al. 2005; Liddle 2004) and multisystemic therapy (MST) (Swensen et al. 2005) and (2) cognitive-behavioral therapies (CBT) (Waldron and Kaminer 2004). Several studies have demonstrated significant improvement among teens with alcohol use disorders who were receiving family-based intervention, group or individual cognitive-behavioral therapy, and therapeutic community interventions (e.g., Waldron and Kaminer 2004; Swensen et al. 2005). All forms of these treatments have substantive differences in intervention design and delivery as well as efficacy evaluation compared with adult alcoholism treatment research (e.g., Brown et al. 2005; Kaminer and Slesnick 2005; Deas et al. 2000). In particular, consideration of youth motivation appears critical in engagement and retention of youth in single-component and complex interventions (e.g., Faw et al. 2005) as well as their continued success following treatment (e.g., Brown and Ramo in press; Kelly et al. 2002). Although limited at this time, evidence is emerging that pharmacologic treatment of co-occurring psychiatric disorders benefits adolescents with alcohol use disorders (e.g., Cornelius et al. 2005). Research on adolescents funded by NIAAA and the National Institute on Drug Abuse has shown that longer adolescent treatments generally show better outcomes. Yet longer (usually complex) treatments can be expensive, and the current health financing system stresses the need for shorter, more cost-effective treatment. This poses a major challenge to alcohol and other drug treatment research today—to identify active ingredients and mechanisms of action of specific components in complex treatments and to determine if such treatments can maintain their effectiveness in reduced forms.

Several models have been proposed to explain adolescent relapse following treatment (e.g., cognitive-behavioral, self-medication) and to predict clinical course after treatment (Brown 2004; Tomlinson et al. 2004). Environmental factors of exposure to substances and use patterns of peers in the immediate social network most consistently emerge as proximal risk factors for adolescent alcohol relapse (e.g., Brown et al. 2005). Personal characteristics including coping skills, self-esteem, and outcome expectancies have been associated with clinical course, as have personality/temperament features linked to disinhibition and negative reactivity (see Brown et al. 2005 for a review). Because a substantial portion of youth relapses are planned rather than unexpected, motivation for sustained abstinence appears to play a critical role in the initial decisions of youth to return to alcohol or other drug involvement after treatment. Although current evidence suggests that developmental factors such as stage of neurocognitive development, psychiatric disorders, and emotional self-regulation play a role in the decisionmaking process regarding relapse, research is needed to explicate the role of each on variability in clinical course.

#### Brief Interventions

A primary function of brief interventions is to motivate people to initiate specific health-related behavior changes. The target of the intervention may be the harmful health behavior itself or consequences of that behavior (e.g., alcohol-related problems). One of the best known of these time-limited strategies (one to five sessions) is motivational enhancement (Miller and Sanchez 1994). This intervention is based on a nonauthoritarian empathic approach that encourages people to take personal responsibility for change, provides objective personalized assessment results on the relative magnitude of the problem behavior, provides explicit advice on the direction to change, and delineates a menu of change options. Brief interventions are flexible in that they can be used to motivate a person to engage in treatment or they can be used as a stand-alone early intervention.

Early evidence on the effectiveness of brief interventions in reducing or eliminating alcohol-related problems in adolescents indicates that they may be effective in reducing both drinking and its consequences (e.g., drunk driving) (Tevyaw and Monti 2004). Recent school-based brief intervention studies suggest that reductions in alcohol use and consequences are mediated by purposeful self-change efforts on the part of teens (e.g., Brown et al. in press) and that expectations of reduction/cessation outcomes may be critical to this change process (e.g., Metrik et al. 2004). One 4-year followup of college freshmen found, however, that reduction in consequences had a lasting effect, whereas reductions in quantity and frequency of alcohol use had washed out by then (e.g., Baer et al. 2001).

## Future Intervention Research

In most adolescent alcoholism treatment studies, developmental criteria have been limited to age and grade as indicators of position along the developmental continuum. However, there is growing recognition of the important contribution that developmentally specific theories, models, and methods can make to the design of innovative and more effective adolescent treatment strategies, outcome measures, and evaluation (Brown 2004).
